# Supplementary material for: A Direct Comparison between the Lateral Magnetophoretic Microseparator and AdnaTest for Isolating Prostate Circulating Tumor Cells
Source: Micromachines (Basel). 2020 Sep 19;11(9):870. doi: 10.3390/mi11090870 (PMC7570110; doi:10.3390/mi11090870)
Supplement: Supplementary file 1 [file micromachines-11-00870-s001.pdf]

Supplementary Materials: A Direct Comparison between the Lateral Magnetophoretic Microseparator and AdnaTest for Isolating Prostate Circulating Tumor Cells

Hyungseok Cho, Jae-Seung Chung and Ki-Ho Han

1. Working Principle of the CTC-μChip

First, the blood samples underwent density gradient centrifugation of RBCs, followed by sequential incubation with anti-EpCAM antibodies and immunomagnetic nanobeads, according to the instructions of the manufacturer. The prepared blood sample and PBS with 0.2% BSA were injected into the sample and buffer inlets of the CTC-μChip, at a flow rate of 2 mL/h (Figure S1a). The width of each inlet was designed to be 500 μm. To enhance the CTC isolation efficiency, the width of the waste and CTC outlets were designed as 800 μm and 200 μm, respectively. To create a high-gradient magnetic field, the width and thickness of the ferromagnetic wires were designed as 70 μm and 40 μm, respectively. The detailed design of the CTC-μChip has been described in previous studies [1,2]. When a uniform external magnetic field is applied to the ferromagnetic wires, the external magnetic field is deformed around the wires, creating a high-gradient magnetic field over the entire area of the microchannel. Then, CTCs labeled with magnetic nanobeads passing over the wires are influenced by the magnetic force, F_m, and hydrodynamic drag force, F_d, generating a lateral magnetic force, F_l, on the CTCs (Figure S1b). Consequently, the magnetized CTCs are forced laterally and flow into the CTC outlet, while normal WBCs flow into the waste outlet (Figure S1a).

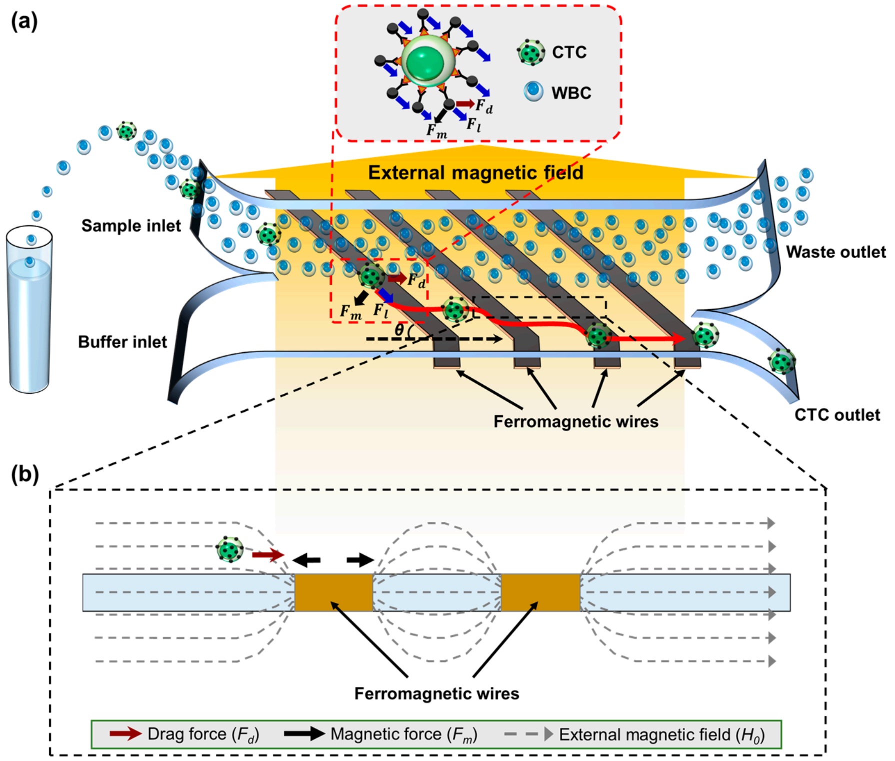

Figure S1. (**a**) Schematic of the working principle of the CTC-μChip and (**b**) an enlarged view of a high-gradient magnetic field created by the ferromagnetic wires, with a uniform external magnetic field.

2. Fabrication of the CTC-μChip

The CTC-μChip comprises a disposable microchannel superstrate and a reusable substrate that can be assembled and disassembled via vacuum pressure. To fabricate the disposable microchannel superstrate, a chromium (Cr, 2,000 Å) layer was first deposited on a 0.7 mm thick glass slide (Borofloat33 Pyrex; Schott) to promote photoresist adhesion (Figure S2a). A 50 μm thick SU-8 channel pattern was created on the Cr-coated glass to make the PDMS replica (Figure S2b). The SU-8 mold was completed by adhesively bonding an acrylic square bar (2 mm × 2 mm) to surround the SU-8 channel pattern (Figure S2c). The liquid-phase PDMS was prepared by mixing resin and a curing agent in a 10:1 ratio (Sylgard 184; Dow Corning), which was then poured into the SU-8 mold and cured for 1 h at 75°C in an oven (Figure S2d). After peeling the PDMS replica off the SU-8 mold, the inlet and outlet reservoirs and the vacuum hole of the PDMS replica were generated using a punch, 1.5 mm in diameter. The final superstrate fabrication step involved bonding the PDMS replica and the 12 μm thick silicone-coated PET release film (release force: 5× g, Shanghai Guangtai Adhesive Products Co.) using oxygen plasma treatment for 60 s at 6.8 W RF power (PDC-32G-2; Harrick Plasma) (Figure S2e).

The reusable substrate includes ferromagnetic wires to generate a high-gradient magnetic field. A titanium/copper/chromium (Ti/Cu/Cr) seed layer was electron beam evaporated onto a 0.7 mm thick bare glass slide (Figure S2f). An SU-8 photoresist was coated with a thickness of 40 μm and patterned for the ferromagnetic wires (Figure S2g). After the patterning process, ferromagnetic permalloy wires (Ni0.8Fe0.2) were electroplated on the patterned side (Figure S2h). Finally, a chemical and mechanical polishing technique was used to flatten the electroplated surface (Figure S2i). For the instrument setup of the CTC-μChip, the disposable microchannel superstrate and the reusable substrate can be assembled and disassembled simply by applying a vacuum (Figure S2j).

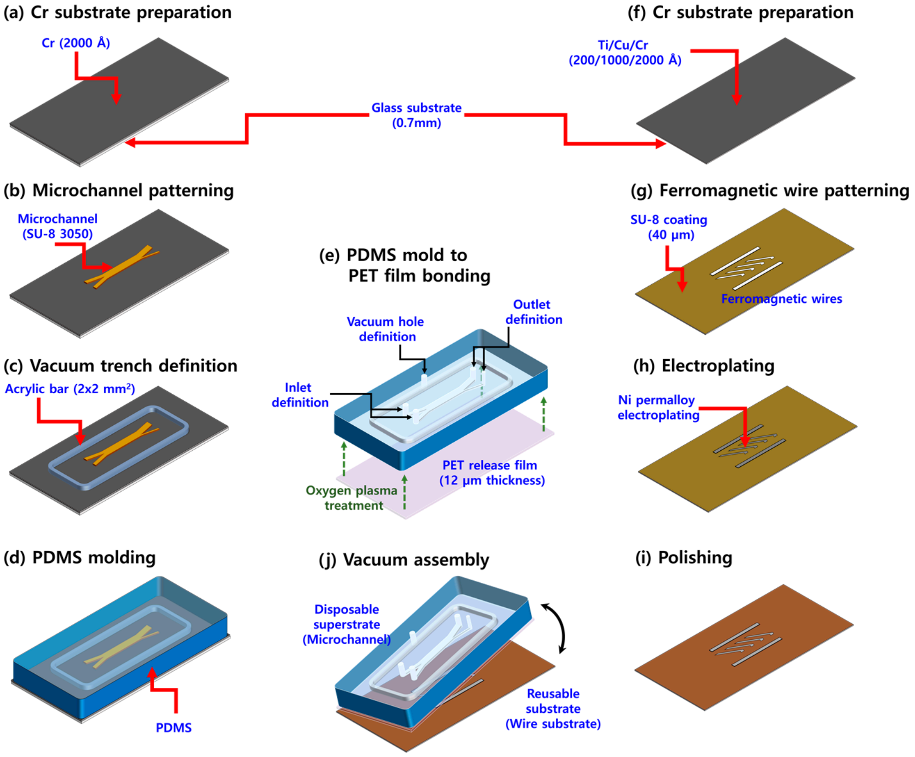

Figure S2. Illustration of the fabrication process of the CTC-μChip. (**a**) Cr layer preparation to fabricate the disposable superstrate, (**b**) patterning 50-μm thick channel mold, (**c**) vacuum trench fabrication, (**d**) PDMS molding process, (**e**) piercing inlet and outlet reservoirs and vacuum hole, then bonding the PET release film to the PDMS replica using oxygen treatment, (**f**) Ti/Cu/Cr seed-layer preparation for the reusable substrate, (**g**) patterning the ferromagnetic wires, (**h**) electroplating the ferromagnetic permalloy, (**i**) polishing the electroplated surface, and (**j**) assembly of the disposable superstrate and reusable substrate.

Table S1. The information and clinical characteristics of enrolled patients with prostate cancer.

**Patient #**	**Age**	**Stage**	**Metastasis**	**Serum PSA level (ng/mL)**	**Gleason score**	**CTC enumeration**	Blood volume (mL)	No. of isolated CTCs per milliliter		No. of isolated WBCs per milliliter		Purity rate (%)	
								CTC-μChip	AdnaTest	CTC-μChip	AdnaTest	CTC-μChip	AdnaTest
**1**	**62**	**T3**	**Bladder**	**10.8**	**9(4+5)**	**-**	5 x2	0.60	N.D.	39.6	N.D.	1.49	N.D.
2	74	T3	No	17.9	7(3+4)	v	4 x4	1.50	0	390	4.75	0.38	0
3	83	T3	N.D.*	25.6	9(4+5)	v	4 x4	3.00	1.00	603	78.5	0.50	1.26
4	69	T4	Bladder	138	7(3+4)	v	5 x4	24.20	1.80	253.2	86.8	8.72	2.03
5	59	T4	Bladder	144	9(4+5)	v	4 x4	30.50	0.50	1530	60.5	1.95	0.82
6	78	mCRPC	Bone	1921	10(5+5)	v	4 x4	5.50	0.75	2693.25	96.75	0.20	0.77
7	65	mHSPC	No	45.3	9(4+5)	-	4 x2	9.00	N.D.	520.5	N.D.	1.70	N.D.
8	76	mCRPC	Bone	203.4	9(4+5)	v	4 x4	13.75	0.50	1028.75	86	1.32	0.58
9	84	mCRPC	Bone	334.4	N.D.	v	4 x4	14.75	0.75	283.5	49.5	4.95	1.49
10	76	mCRPC	Bone	152.2	10(5+5)	-	5 x2	18.60	N.D.	897	N.D.	2.03	N.D.
11	80	mCRPC	Bone	301.8	8(4+4)	v	4 x4	19.50	0.75	347.25	79	5.31	0.94
12	67	mCRPC	Bone	23.9	10(5+5)	-	4 x2	20.75	N.D.	142.25	N.D.	12.73	N.D.
13	78	mCRPC	Bone	49.81	9(4+5)	v	4 x4	29.00	0.50	436	81.5	6.24	0.61
14	79	mHSPC	Bladder	5000	8(4+4)	v	4 x4	40.75	10.50	313.5	69.5	11.50	13.13

* N.D.: No data.

3. Protocols for mRNA Extraction and cDNA Synthesis

After the CTCs were isolated, the cells were lysed using 300 μL of lysis/binding buffer (Dynabeads mRNA Direct Kit, Invitrogen), with gentle pipette mixing until the lysis was completed. The mRNA sample was extracted by adding 20 μL of washed 2.8-μm diameter magnetic beads (Dynabeads Oligo(dT)25, Invitrogen) to the lysed sample, as these beads specifically bind to the poly-A tails of mRNA. After 10 min of mixing and incubation, the samples were placed on a magnet for 2 min to collect the oligo-dT magnetic beads with mRNA. Two washing step sequences were used after the oligo-dT magnetic bead collection according to the manufacturer's protocol, which used washing buffer A two times and washing buffer B three times. The mRNA sample was eluted from the magnetic beads using 10 μL of elution buffer (10 mM Tris-HCL pH 7.5), which was then incubated at 75°C for 2 min. The tube was again placed on the magnet and the attached template was transferred to a new PCR tube for the cDNA synthesis procedure. The eluted mRNA templates were directly synthesized using 10 μL of 0.1% diethylpyrocarbonate (DEPC)-dissolved AccuPower CycleScript RT PreMix (dT20) (Bioneer). After adding the mRNA template to the premixed RT solution, the reaction mixture was incubated at 42°C for 90 min in a thermal cycler, with a terminal RNase inactivation step at 95°C for 5 min.

4. Protocol for Multiplex PCR Pre-Amplification

The endpoint multiplex PCR method was used to evaluate the primer sets for cancer-related genes (AR, AR-V7, PSMA, KRT-19, and CD45). The pre-amplification primer sets are listed at Table S2, and the cDNA were synthesized using the S3 procedures (above). For the pre-amplification procedure, 5 μL of cDNA template were mixed with the multiplex PCR supermix (AccuPower HotStart PCR PreMix; Bioneer) and the forward and reverse primers (10 pmol/μL, final concentration: 100 nM) according to the manufacturer's protocol. For the multiplex PCR, the 5 gene primer sets were added to the same amount of mixed cDNA templates in the multiplex PCR premix (AccuPower Multiplex PCR PreMix; Bioneer), according to the manufacturer's protocol. The PCR conditions (ramp rate: 4°C/s) were denaturing at 95°C for 10 min; then 18 cycles of 95°C for 30 s, 56°C for 1 min, and 72°C for 30 s; and a final extension at 72°C for 10 min.

Table S2. Pre-amplification primer sequences with product sizes.

Gene	Nucleotide sequence	Primer sequence	Product size (bp)
AR (Androgen receptor)	NM_000044.4<br>NM_001011645.3	F : 5′-TCTCTCAAGAGTTTGGATGGC-3′<br>R : 5′-ACTGGGTGTGGAAATAGATGG-3′	390
AR-V7 (Androgen receptor variant 7)	NM_001348061.1<br>NM_001348064.1	F : 5′-GTGCGCCAGCAGAAATGA-3′<br>R : ATGCCAAGCCACATTACAGG-3′	440
PSMA (Prostate specific membrane antigen)	NM_004476.2<br>NM_001014986.2<br>NM_001193471.2<br>NM_001193472.2<br>NM_001351236.1	F : 5′-AGCATTTTTGGATGAATTGAAAGC-3′<br>R : 5′-TGGGTAACCTGGTGTGAGA-3′	643
KRT19 (Cytokeratin 19)	NM_002276.4	F : 5′ – CGACTACAGCCACTACTACAC – 3′<br>R : 5′ – CACTATCAGCTCGCACATCG – 3′	695
CD45 (Protein tyrosine phosphatase, receptor type, C)	NM_002838.4<br>NM_080921.3	F : 5′ – TGTTGTAAAGATCAACCAGCAC – 3′<br>R : 5′ - CTGTGTCCTCCAGCTCCTA – 3′	544

5. Protocol for ddPCR

After pre-amplification, the pre-amplified samples were diluted in a ratio of 1:10 with deionized water. The diluted template (5 µL) was mixed with 10 µL of EvaGreen Supermix (QX200 ddPCR EvaGreen® Supermix, Bio-Rad) and the target gene primer sets (10 pmol/µL, final concentration: 100 nM). The target gene primer sequences and product sizes are listed in Table S3.

To generate the droplet, the prepared PCR mixture was loaded into a droplet-generating cartridge (DG8™ Cartridges for QX100™/QX200™ Droplet Generator, Bio-Rad) with 70 µL of the droplet generation oil (Droplet Generation Oil for EvaGreen, Bio-Rad). The cartridge was then loaded into the droplet generator (QX200™ Droplet Generator, Bio-Rad). Before the PCR procedure, the generated droplets were transferred to 96-well PCR plates, which were then thermally sealed to prevent evaporation (PX1™ PCR Plate Sealer, Bio-Rad). The sealed PCR plate was then loaded into the PCR cycler (GeneAmp® PCR System 9700, Applied Biosystems). The PCR cycling steps (ramp rate: 2°C/s) were 5 min at 95°C for enzyme activation and followed by 40 cycles of 95°C for 30 s (denaturation) and 56°C for 1 min (annealing plus extension). After the cycling steps, the signal stabilization sequence was 4°C for 5 min and 90°C for 5 min, with optional holding at 12°C until the reading process (QX200™ Droplet Reader, Bio-Rad).

Table S3. Primer sequences and product sizes of the target gene.

Gene	Nucleotide sequence	Primer sequence	Product size (bp)
AR (Androgen receptor)	NM_000044.4<br>NM_001011645.3	F : 5′-CAGCCTATTGCGAGAGAGCTG-3′<br>R : 5′-GAAAGGATCTTGGGCACTTGC-3′	125
AR-V7 (Androgen receptor variant 7)	NM_001348061.1<br>NM_001348064.1	F : 5′- CCATCTTGTCGTCTTCGGAAATGTTA-3′<br>R : 5′- TTTGAATGAGGCAAGTCAGCCTTTCT-3′	125
PSMA (Prostate specific membrane antigen)	NM_004476.2<br>NM_001014986.2<br>NM_001193471.2<br>NM_001193472.2<br>NM_001351236.1	F : 5′-ATGAATTGAAAGCTGAGAACATCAAGA -3′<br>R : 5′-GGGATGAGTCTTATTTGGGTAGGAC-3′	188
KRT19 (Cytokeratin 19)	NM_002276.4	F : 5'-TTTGAGACGGAACAGGCTCT-3'<br>R : 5'-AATCCACCTCCACACTGACC-3'	211
CD45 (Protein tyrosine phosphatase, receptor type, C)	NM_002838.4<br>NM_080921.3	F : 5′-CTTCAGTGGTCCCATTGTGGTG-3′<br>R : 5′-CCACTTTGTTCTCGGCTTCCAG-3′	117

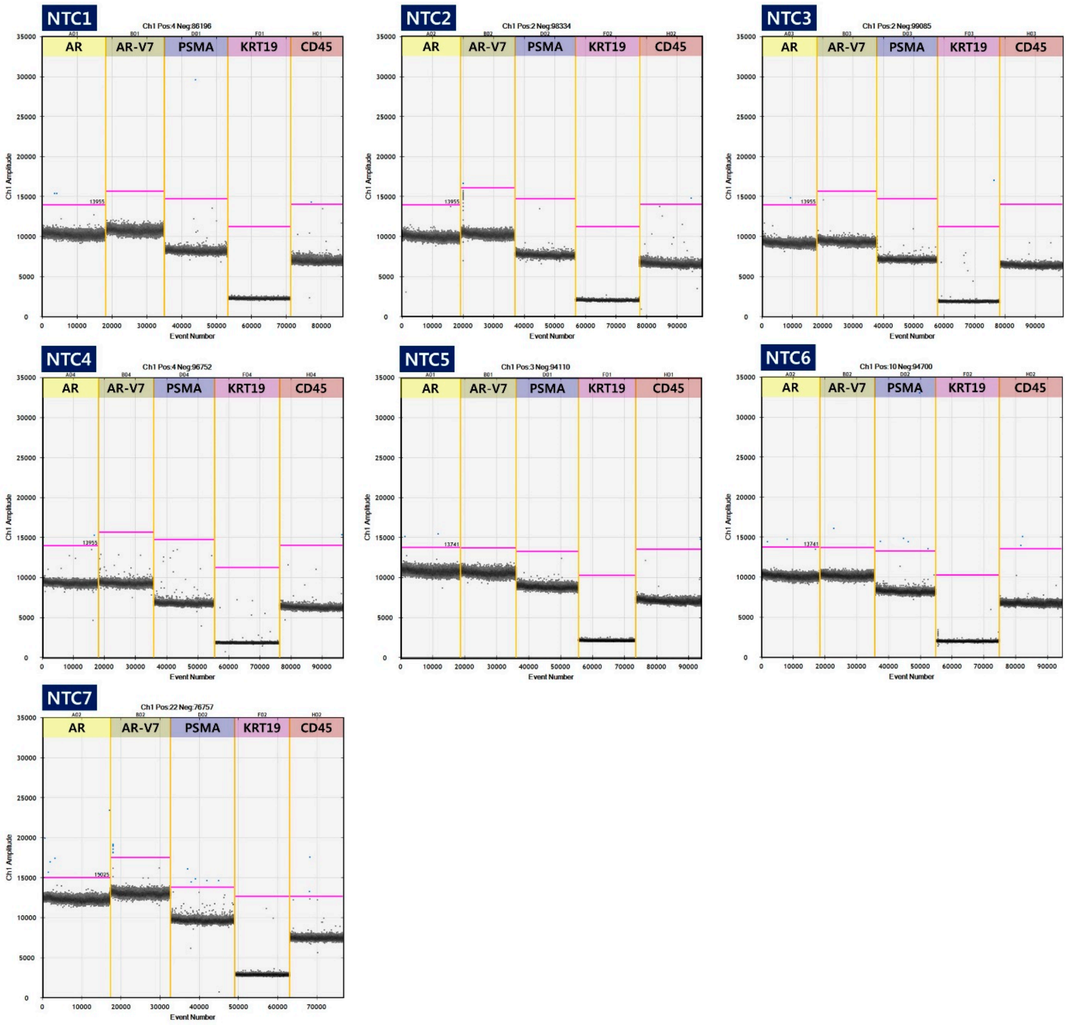

Figure S3. Droplet fluorescence amplitudes in ddPCR assays with seven NTC samples to determine the fluorescence thresholds of the selected five genes.

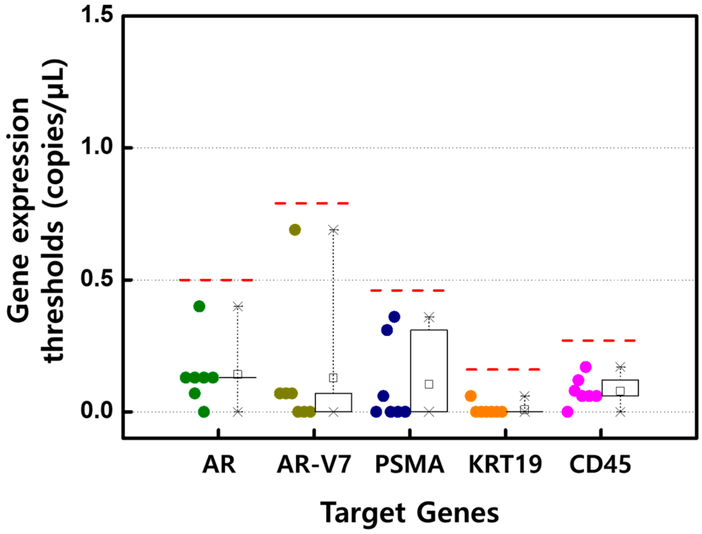

Figure S4. The gene expression thresholds of the selected five genes were determined as 0.5 copies/μL at AR, 0.79 copies/μL at AR-V7, 0.46 copies/μL at PSMA, 0.16 copies/μL at KRT19, and 0.27 copies/μL at CD45.

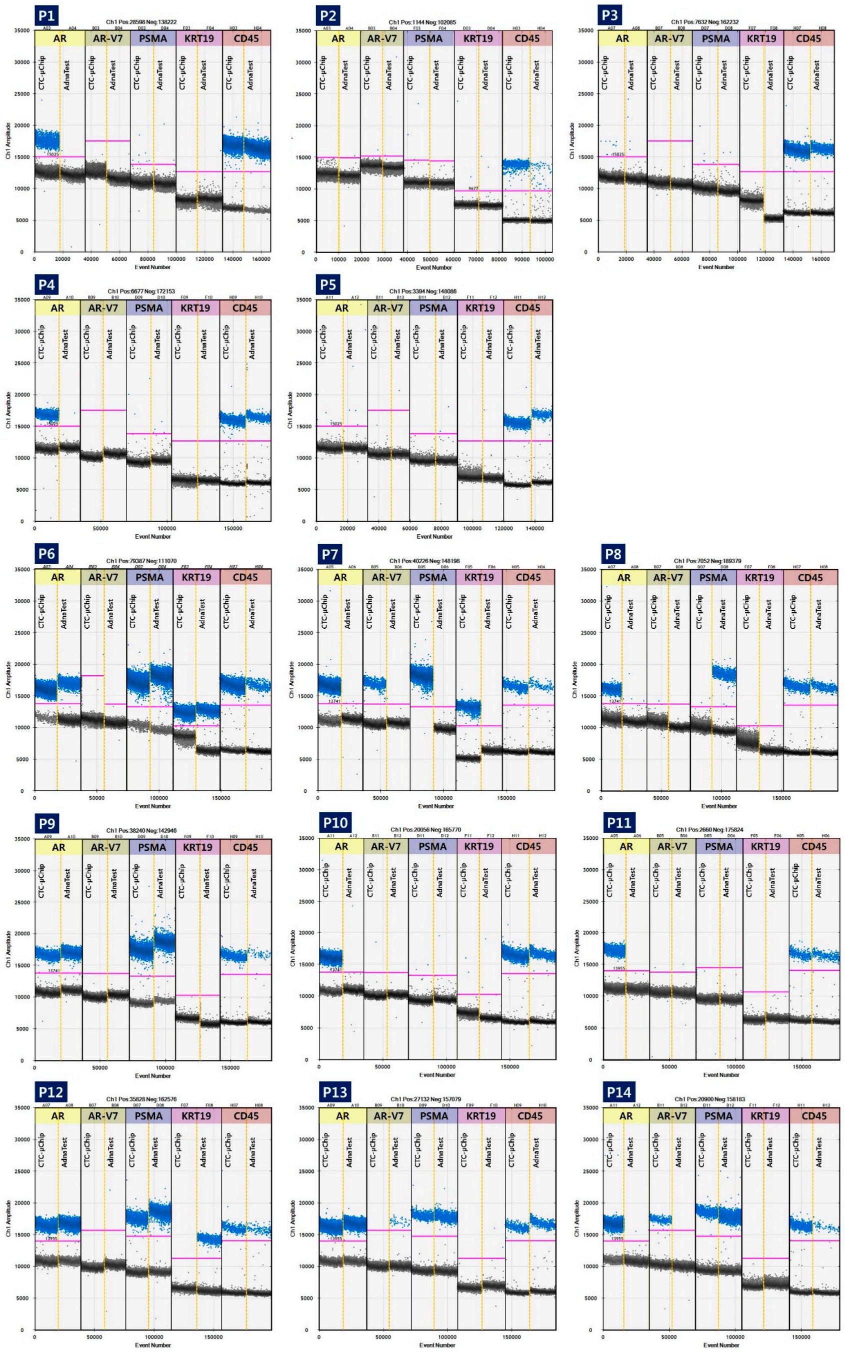

Figure S5. Droplet fluorescence amplitudes in ddPCR assays of the five genes in CTCs isolated from patients (*n* = 14) with primary (P1 to P5) and metastatic (P6 to P14) prostate cancer using the CTC-μChip and the AdnaTest.

References

1. Cho, H.; Kim, J.; Jeon, C.-W.; Han, K.-H. A disposable microfluidic device with a reusable magnetophoretic functional substrate for isolation of circulating tumor cells. *Lab on a Chip* **2017**, *17*, 4113–4123.
2. Kim, S.; Han, S.-I.; Park, M.-J.; Jeon, C.-W.; Joo, Y.-D.; Choi, I.-H.; Han, K.-H. Circulating tumor cell microseparator based on lateral magnetophoresis and immunomagnetic nanobeads. *Analy. Chem.* **2013**, *85*, 2779–2786.
